# A novel nomogram to predict futile recanalization in patients with acute ischemic stroke undergoing mechanical thrombectomy

**DOI:** 10.3389/fneur.2024.1367950

**Published:** 2024-03-22

**Authors:** Cheng-cai Lai, Yin-dan Yao, Xia Li, Ao-fei Liu, Chen Li, Yun-e Liu, Chang-chun Jiang, Ying-ying Zhang, Min Jin, Jin Lv, Wei-jian Jiang

**Affiliations:** ^1^The PLA Rocket Force Characteristic Medical Center, Beijing, China; ^2^Department of Pharmaceutical Sciences, Beijing Institute of Radiation Medicine, Beijing, China; ^3^Department of Neurology, Ningbo No.2 Hospital, Ningbo, Zhejiang, China; ^4^Department of Neurology, Baotou Center Hospital, Baotou, China; ^5^Neurointerventional Medical Center of Inner Mongolia Medical University, Hohhot, Inner Mongolia, China; ^6^Institute of Cerebrovascular Disease in Inner Mongolia, Hohhot, Inner Mongolia, China

**Keywords:** acute ischemic stroke, futile recanalization, mechanical thrombectomy, nomogram, predict

## Abstract

**Background and objective:**

Futile recanalization (FR) is defined as patients with acute ischemic stroke (AIS) due to large vessel occlusion who still exhibits functional dependence although undergoing successful mechanical thrombectomy (MT). We aimed to develop and validate a simple nomogram for predicting the probability of FR after MT treatment in AIS patients.

**Methods:**

Clinical data of AIS patients in the Jrecan clinical trial in China from March 2018 to June 2019 were collected as the derivation set (*n* = 162). Meanwhile, clinical data of AIS patients who underwent MT in Baotou Central Hospital and Ningbo No.2 Hospital from 2019 to 2021 were collected as the validation set (*n* = 170). Multivariate logistic regression analysis was performed for all variables that had *p* < 0.2 in the univariate analysis in the derivation set. The independent risk factors of FR were further screened out and a nomogram was constructed. The performance of the nomogram was analyzed in the derivation and validation set using C-index, calibration plots, and decision curves.

**Results:**

No significant difference in FR rate was detected between the derivation set and the validation set [88/162 (54.32%) and 82/170 (48.23%), *p* = 0.267]. Multivariate logistic regression analysis showed that age ≥ 65 years old (OR = 2.096, 95%CI 1.024–4.289, *p* = 0.043), systolic blood pressure (SBP) ≥ 180 mmHg (OR = 5.624, 95%CI 1.141–27.717, *p* = 0.034), onset to recanalization time (OTR) ≥ 453 min (OR = 2.759, 95%CI 1.323–5.754, *p* = 0.007), 24 h intracerebral hemorrhage (ICH; OR = 4.029, 95%CI 1.844 ~ 8.803, *p* < 0.001) were independent risk factors for FR. The C-index of the nomogram of the derivation set and the verification set were 0.739 (95%CI 0.662~0.816) and 0.703 (95%CI 0.621~0.785), respectively.

**Conclusion:**

The nomogram composed of age, SBP, OTR, and 24 h ICH can effectively predict the probability of FR after MT in AIS patients.

## Introduction

At present, the main therapeutic methods for AIS patients in the time window are intravenous thrombolysis and mechanical thrombolysis (MT). The efficacy and safety of MT in the treatment of acute large vessel occlusive stroke (LVOS) have been proven, and it is the first-line treatment strategy recommended by current guidelines (1). However, there is still evidence that about 50% of patients have poor prognosis after MT (2). In order to maximize the benefit of MT treatment for more patients, more and more researchers are committed to analyzing preoperative and perioperative characteristics of patients to explore the risk factors affecting poor prognosis. Researchers have established several clinical prediction models and scoring systems, including THREIVE, PRE and IER-START nomogram, to accurately predict and evaluate the effect of MT treatment (3). Although this kind of prediction model and rating scale have certain forecasting efficiency, there are still shortcomings, which need to be further improved and verified. Some AIS patients could not achieve functional independence after successful MT therapy was named as futile recanalization (FR). Many studies (2, 4–6) are committed to building FR risk prediction models for acute LVOS patients after MT, but most of them are single-sample and retrospective studies, and there is still a lack of prospective, multicenter, and randomized studies in clinical practice.

This study was based on a reanalysis of data from a multicenter randomized controlled trial (RCT). By analyzing risk-related factors affecting FR, a nomogram was established to predict the probability of FR on AIS patients after MT. On the basis of previous studies, the risk factors of age, systolic blood pressure (SBP), onset to recanalization time (OTR), and 24 h intracerebral hemorrhage (ICH) were subdivided to better construct a risk prediction model for FR.

## Materials and methods

### Study design and population

The derivation cohorts in this study were enrolled in the Jrecan trial between 1 March 2018 and 30 June 2019. The Jrecan trial, a prospective, multicenter, randomized, non-inferior clinical trial, was designed to verify the safety and efficacy of the Jrecan Flow Reconstruction Device manufactured by Hunan Ruikang Technologies Co., Ltd. for endovascular treatment of acute ischemic stroke, and registered with Chinese Clinical Trial Registry (NO. ChiCTR-TOC-17013822, http://www.chictr.org.cn/showproj.aspx?proj=23396). The trial involved 16 high-capacity stroke centers in China and was approved by PLA Rocket Force General Hospital Ethics Committee (NO. X2017008). The validation cohorts in this study were from Baotou central hospital (March 2019–December 2021) and Ningbo No.2 Hospital (February 2019–December 2021), with 76 and 94 cases, respectively.

The studies were included if they met the following criteria: (1) Aged ≥18 years old; (2) AIS: within 8 h; (3) baseline admission National Institute of Health Stroke Scale (NIHSS) score ≥ 6；(4) modified Rankin Scale (mRS) score before onset ≤ 2; (5) angiography confirmed intracranial internal carotid artery, middle cerebral artery (MCA) trunk acute obstruction in patients; (6) The subject (or his guardian) agreed to participate in the study and signed an informed consent form.

The exclusion criteria were as follows: (1) CT or MR showed evidence of intracranial hemorrhage, or is known to have bleeding tendency; (2) CT showed low-density lesions more than 1/3 of the middle cerebral artery area, or ASPECTS < 7; (3) DSA showed carotid artery dissection, complete occlusion of cervical carotid artery or vasculitis; (4) DSA prompted serious tortuous path blood vessels, blood flow reconstruction device is difficult to reach the target location; (5) DSA angiography prompted an acute blockage of 2 vessels; (6) had a history of stroke within 3 months; (7) had heart, lung, liver and kidney failure or other serious diseases (such as brain tumors, cerebral arteriovenous malformations, systemic infections, active disseminated intravascular coagulation, myocardial infarction within 12 months before enrollment, A serious history of mental illness); (8) Pregnant or lactating women; (9) was known to be severely allergic to contrast media; (10) were participating in other drug or device research; (11) Life expectancy was less than 90 days.

### Data collection

The epidemiological information and clinical data of the admitted patients were the same as in our previous studies (7). In this study, clinical data of patients were collected as follows: (1) Demographic information: age and sex; (2) Previous medical history: hypertension, diabetes, coronary heart disease, atrial fibrillation, previous stroke history; (3) Baseline data: preoperative mRS Score, blood glucose, blood pressure, preoperative NIHSS score, ASPECT score, whether intravenous thrombolysis was performed; (4) Intraoperative and postoperative: first pass effect, anesthesia method, lesion site, residual severe stenosis degree, remedial treatment, anticoagulation therapy, recanalization time; (5) Complications: 24 h ICH, 24 h sICH, survival status. See Table 1 for details.

**Table 1:** Univariate analysis of the factor associating with futile recanalization vs. successful recanalization in training set and validation set of patients after mechanical thrombectomy.

Variables	Statistics	Derivation set (*n* = 162)		Validation set (*n* = 170)	
		FR	ER	FR	ER
		(mRS ≥ 3, *n* = 88)	(mRS ≤ 2, *n* = 74)	(mRS ≥ 3, *n* = 82)	(mRS ≤ 2, *n* = 88)
**Demographics**					
Age	Median, IQR	67 (61~74)	64 (52~70.25)	70.5 (61~77)	64 (55~72)
	≥65	69.3% (61/88)	47.3% (35/74)	67.1% (55/82)	46.6% (41/88)
	<65	30.7% (27/88)	52.7% (39/74)	32.9% (27/82)	53.4% (47/88)
	*P*-value	0.004		0.007	
Sex	Male	55.7% (49/88)	62.2% (46/74)	50.0% (41/82)	55.7% (49/88)
	Female	44.3% (39/88)	37.8% (28/74)	50.0% (41/82)	44.3% (39/88)
	*P*-value	0.404		0.458	
**Medical history**					
Hypertension	Yes	64.8% (57/88)	51.4% (38/74)	67.1% (55/82)	51.1% (45/88)
	No	35.2% (31/88)	48.6% (36/74)	32.9% (27/82)	48.9% (43/88)
	*P*-value	0.084		0.035	
Diabetes	Yes	25.0% (22/88)	21.6% (16/74)	25.6% (21/82)	13.6% (12/88)
	No	75.0% (66/88)	78.4% (58/74)	74.4% (61/82)	86.4% (76/88)
	*P*-value	0.613		0.049	
Coronary heart disease	Yes	19.3% (17/88)	14.9% (11/74)	20.7% (17/82)	14.8% (13/88)
	No	80.7% (71/88)	85.1% (63/74)	79.3% (65/82)	85.2% (75/88)
	*P*-value	0.455		0.308	
Atrial fibrillation	Yes	53.4% (47/88)	44.6% (33/74)	56.1% (46/82)	37.5% (33/88)
	No	46.6% (41/88)	55.4% (41/74)	43.9% (36/82)	62.5% (55/88)
	*P*-value	0.264		0.015	
Previous stroke	Yes	12.5% (11/88)	10.8% (8/74)	19.5% (16/82)	13.6% (12/88)
	No	87.5% (77/88)	89.2% (66/74)	80.5% (66/82)	86.4% (76/88)
	*P*-value	0.739		0.302	
**Baseline data**					
Pre-operation mRS	=0	93.2% (82/88)	95.9% (71/74)	69.5% (57/82)	93.2% (82/88)
	≠0	6.8% (6/88)	4.1% (3/74)	30.5% (25/82)	6.8% (6/88)
	*P*-value	0.444		<0.001	
Glucose	Median, IQR	7.51 (6.02 ~ 9.68)	6.55 (5.82 ~ 8.72)	7.84 (6.80 ~ 9.87)	6.5 (5.6 ~ 7.53)
	≥6.91 mmol/L	59.1% (52/88)	39.2% (29/74)	71.4% (50/70)	35.8% (29/81)
	<6.91 mmol/L	40.9% (36/88)	60.8% (45/74)	28.6% (20/70)	64.2% (52/81)
	*P*-value	0.012		<0.001	
Systolic pressure	Median, IQR	138 (124 ~ 160)	138.5(122 ~ 156)	145 (127 ~ 164)	142 (130 ~ 160)
	≥180 mmHg	13.6% (12/88)	2.7% (2/74)	10.7% (8/75)	12.6% (11/87)
	<180 mmHg	86.4% (76/88)	97.3% (72/74)	89.3% (67/75)	87.4% (76/87)
	*P*-value	0.014		0.697	
Diastolic pressure	Median, IQR	82 (73 ~ 89)	82 (78 ~ 92)	88 (77 ~ 95)	89 (76 ~ 100)
	≥101 mmHg	12.5% (11/88)	8.1% (6/74)	14.7% (11/75)	23.0% (20/87)
	<101 mmHg	87.5% (77/88)	91.9% (68/74)	85.3% (64/75)	77.0% (67/87)
	*P*-value	0.364		0.179	
Initial NIHSS	Median, IQR	14 (12~19)	14 (11~17)	19 (14~26)	14 (10.2~17.8)
	≥19	28.4% (25/88)	17.6% (13/74)	51.2% (942/82)	20.5% (18/88)
	<19	71.6% (63/88)	82.4% (61/74)	48.8% (40/82)	79.5% (70/88)
	*P*-value	0.105		<0.001	
ASPECT	10	27.3% (24/88)	43.2% (32/74)	25.6% (21/82)	21.6% (19/88)
	9	15.9% (14/88)	16.2% (12/74)	22.0% (18/82)	31.8% (28/88)
	8	28.4% (25/88)	27.0% (20/74)	32.9% (27/82)	34.1% (30/88)
	7	28.4% (25/88)	13.5% (10/74)	19.5% (16/82)	12.5% (11/88)
	*P*-value	0.068		0.369	
TOAST	Atheroma	15.9% (14/88)	21.6% (16/74)	53.7% (44/82)	48.9% (43/88)
	Cardioembolic	84.1% (74/88)	78.4% (58/74)	35.4% (29/82)	44.3% (39/88)
	Dissection	--	--	3.7% (3/82)	1.1% (1/88)
	Others	--	--	7.3% (6/82)	5.7% (5/88)
	*P*-value	0.351		0.500	
Intravenous thrombolysis	Yes	30.7% (27/88)	33.8% (25/74)	36.6% (30/82)	31.8% (28/88)
	No	69.3% (61/88)	66.2% (49/74)	63.4% (52/82)	68.2% (60/88)
	*P*-value	0.674		0.512	
**Procedure aspect**					
First pass effect	Yes	39.8% (35/88)	40.5% (30/74)	40.2% (33/82)	48.9% (43/88)
	No	60.2% (53/88)	59.5% (44/74)	59.8% (49/82)	51.1% (45/88)
	*P*-value	0.921		0.259	
Anesthesia methods	GA	42.0% (37/88)	55.4% (41/74)	61.0% (50/82)	40.9% (36/88)
	Local	58.0% (51/88)	44.6% (33/74)	39.0% (32/82)	59.1% (52/88)
	*P*-value	0.090		0.009	
Location of lesions	M1	65.9% (58/88)	78.4% (58/74)	52.4% (43/82)	59.1% (52/88)
	ICA	34.1% (30/88)	21.6% (16/74)	47.6% (39/82)	40.9% (36/88)
	*P*-value	0.080		0.383	
Residual severe stenosis	Yes	11.4% (10/88)	20.3% (15/74)	12.2% (10/82)	20.5% (18/88)
	No	88.6% (78/88)	79.7% (59/74)	87.8% (72/82)	79.5% (70/88)
	*P*-value	0.118		0.147	
Rescue therapy	Yes	21.6% (19/88)	20.3% (15/74)	17.1% (14/82)	14.8% (13/88)
	No	78.4% (69/88)	79.7% (59/74)	82.9% (68/82)	85.2% (75/88)
	*P*-value	0.837		0.682	
Anticoagulant therapy	Yes	52.3% (46/88)	60.8% (45/74)	11.0% (9/82)	9.1% (8/88)
	No	47.7% (42/88)	39.2% (29/74)	89.0% (73/82)	90.9% (80/88)
	*P*-value	0.275		0.682	
OTR	Median, IQR	408 (315~497)	410.5 (324~480)	378.5 (257~463)	362.0 (254~435)
	≥453 min	44.3% (39/88)	29.7% (22/74)	25.6% (21/82)	23.0% (20/87)
	<453 min	55.7% (49/88)	70.3% (52/74)	74.4% (61/82)	77.0% (67/87)
	*P*-value	0.056		0.691	
**Complications**					
24 h ICH	Yes	44.6% (37/83)	18.9% (14/74)	43.8% (35/80)	20.5% (18/88)
	No	55.4% (46/83)	81.1% (60/74)	56.3% (45/80)	79.5% (70/88)
	*P*-value	0.001		0.001	
24 h sICH	Yes	9.6% (8/83)	0% (0/74)	24.7% (20/81)	1.1% (1/88)
	No	90.4% (75/83)	100.0% (74/74)	75.3% (61/81)	98.9% (87/88)
	*P*-value	0.006		<0.001	
Survival status	Survival	61.4% (54/88)	100% (74/74)	74.4% (61/82)	100% (88/88)
	Death	38.6% (34/88)	0% (0/74)	25.6% (21/82)	0% (0/82)
	*P*-value	<0.001		<0.001	

FR indicates futile recanalization; ER indicates effective recanalization; mRS indicates modified Rankin Scale; NIHSS indicates national Institute of Health Stroke Scale; ASPECT indicates Alberta Stroke Program Early CT Score; ICH indicates intracranial hemorrhage; sICH indicates symptomatic intracranial hemorrhage; OTR indicates time from symptom onset to recanalization.

### Definition of clinical outcomes

Effective recanalization (ER) was defined as modified thrombolysis in cerebral infarction (mTICI) scores of 2b or higher and associated rates of functional independence or good functional outcomes, defined as mRS ≤ 2 at 90 days. FR was defined as the level of modified TICI (mTICI) 2b to 3, despite successful recanalization, modified Rankin Scale (mRS) scores ≥3 still appeared after 90d (4, 8). Symptomatic intracranial hemorrhage (sICH) was defined as an increase of ≥4 in the NIHSS score within 24 h or death. Early neurological deterioration (END) was defined as increase in ≥4 points of the NIHSS between admission and 24 h.

### Statistical analysis

All data analyses were performed using SPSS software (version 22.0, IBM Corporation, Armonk, New York, United States) and R version 4.2.1 software (http://www.R-project.org, foundation for statistical computing, Vienna, Austria). The normally distributed continuous data were expressed as mean ± standard deviation ( $\bar{X}$ ± s), and the T-test was used for comparison between groups. Non-normally distributed continuous variables were expressed as median (quartile) [M (Q1, Q3)], and Mann Whitney U test was used for comparison between groups. Count data were expressed in cases (percentages) and comparisons between groups were performed using either the x^2^ test or Fisher’s exact probability test. Multivariate logistic regression analysis was used to determine significant predictors of FR, and a nomogram was established. The performance of the nomogram was verified in the modeling group and the verification group by C-index, calibration chart, Hosmer Lemeshow test and decision curve analysis (DCA), respectively. C-index were used to measure discrimination; The calibration plot described the degree of fit between the occurrence of the actual invalid reconnection and the nomogram predicted invalid reconnection; DCA to present the net return at various threshold probabilities.

## Results

### Baseline features of the participants

**Figure 1:**
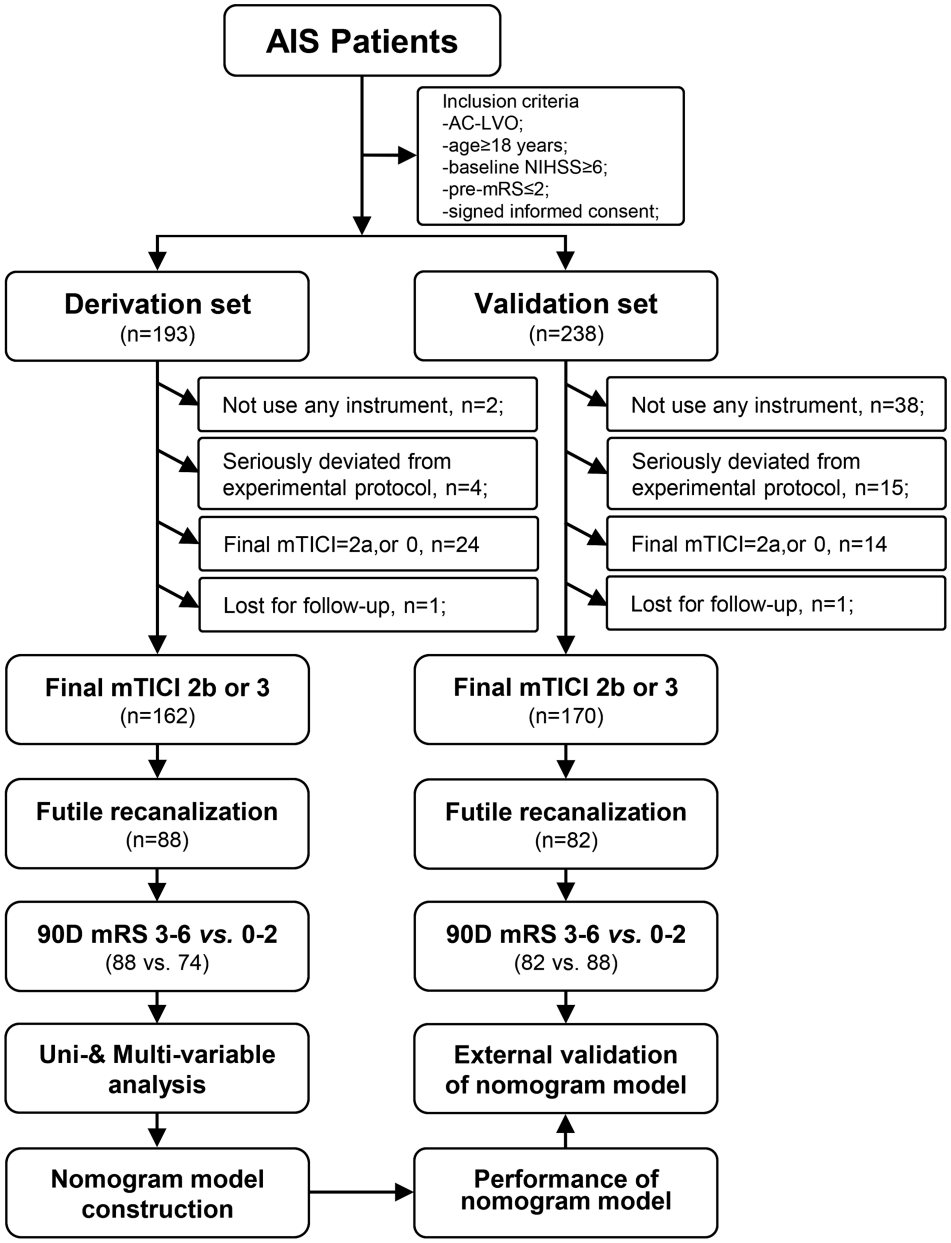
Selection criteria and workflow of the study.

The derivation set was based on RCT data from Jrecan and initially included 193 patients. 2 cases did not use any instrument, 4 cases were seriously deviated from experimental protocol, 1 case was lost for follow-up, and 24 cases had a mTICI of 2a or 0 after opening. A total of 162 eligible patients were finally included in the derivation set (Figure 1). The median age of the patients was 66 years old (56–74), and 58.9% were male. Other clinical information is shown in Supplementary Table 1. In the derivation set, there were 88 cases of FR and 74 cases of ER, with FR accounting for 54.32%.

The validation set initially included 238 people, 38 cases did not use any instrument, 15 cases were seriously deviated from experimental protocol, 1 case was lost for follow-up, and 14 cases had mTICI of 2a or 0 after MT. Therefore, a total of 170 people were eventually included in the validation set (Figure 1). The median age of patients was 67 years (59–75), the proportion of males was 52.9%, and other clinical information was shown in Supplementary Table 1. In the validation set, 82 cases were FR and 88 cases were ER, with FR accounting for 48.24%.

**Figure 2:**
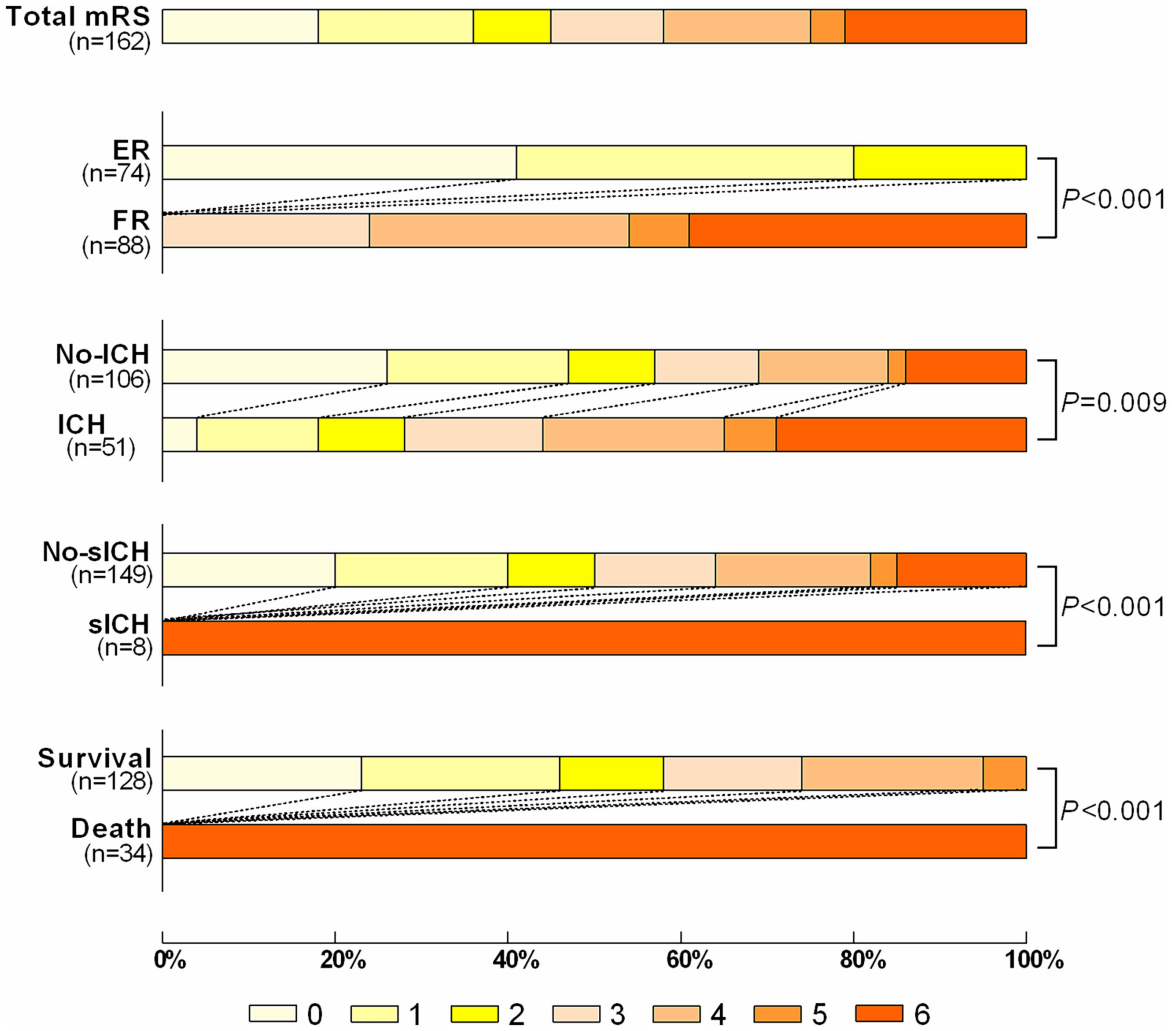
The distribution of the modified Rankin Scale (mRS) of patients with ER vs. FR; ICH vs. no-ICH; sICH vs. no-sICH; Survival vs. Death. ER indicates effective recanalization; FR indicates futile recanalization; ICH indicates intracranial hemorrhage; sICH indicates symptomatic intracranial hemorrhage.

There was no significant statistical difference in FR rates between the derivation set and validation set [88/162 (54.32%) and 82/170 (48.23%), *p* = 0.267] (Supplementary Table 1). The 90d mRS Scores of the patients in the derivation set were shown in Figure 2, including ER group/FR group, ICH group/No-ICH group, sICH group/No-sICH group, and survival group/death group, with significant statistical differences in each group.

### Risk factors selection

The baseline characteristics of ER and FR in the derivation set and the results of univariate analysis are listed in Table 1. To prevent missing meaningful variables, factors with *p* < 0.2 were shown as potential predictors of FR in the derivation set. The predictors included in multivariate analysis were Age (*p* = 0.004), Hypertension (*p* = 0.084), Glucose (*p* = 0.012), Systolic pressure (*p* = 0.014), and Initial NIHSS (*p* = 0.105), ASPECT (*p* = 0.068), Anesthesia methods (*p* = 0.090), Location of lesions (*p* = 0.080), Residual severe stenosis (*p* = 0.118), OTR (*p* = 0.056), 24 h ICH (*p* = 0.001).

The risk factors of FR in the derivation set based on the results of multivariate Logistic analysis are shown in Table 2. The results showed that Age ≥ 65 years old (OR = 2.096, 95%CI 1.024–4.289, *p* = 0.043), SBP ≥ 180 mmHg (OR = 5.624, 95%CI 1.141–27.717, *p* = 0.034), OTR ≥ 453 min (OR = 2.759, 95%CI 1.323~5.754, *p* = 0.007), 24 h ICH (OR = 4.029, 95%CI 1.844~8.803, *p* < 0.001) was an independent risk factor for FR.

**Table 2:** Multivariable analysis of risk factors associated with futile recanalization in patients after mechanical thrombectomy.

Variables	Derivation set (*n* = 163)	
	OR (95%CI)	*P-*value
Age ≥ 65 years	2.096 (1.024~4.289)	0.043
SBP ≥ 180 mmHg	5.624 (1.141~27.717)	0.034
OTR ≥ 453 min	2.759 (1.323~5.754)	0.007
24 h ICH	4.029 (1.844~8.803)	<0.001

SBP indicates systolic blood pressure; OTR indicates time from symptom onset to recanalization; ICH indicates intracranial hemorrhage.

### Nomogram construction and performance validation

**Figure 3:**
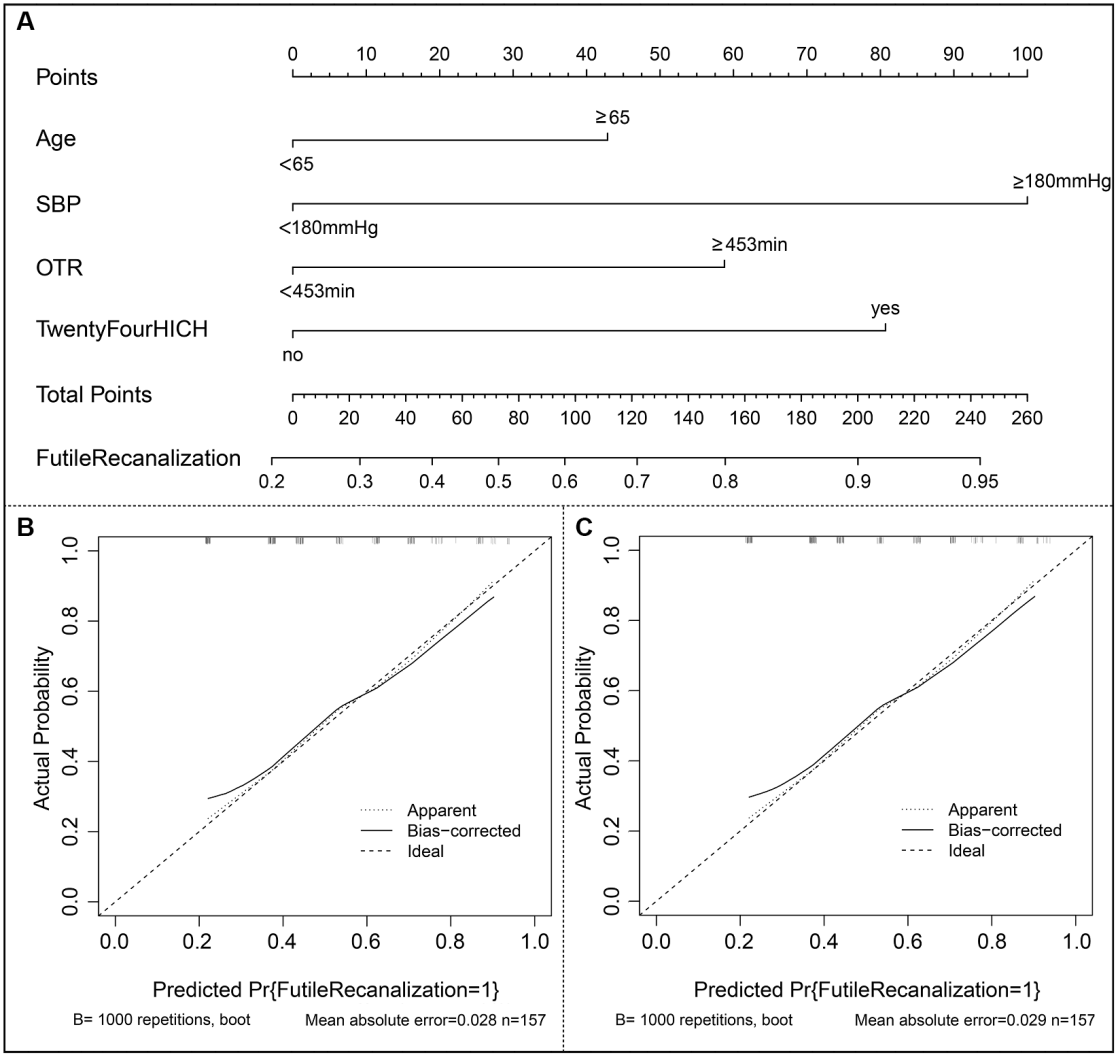
Construction of nomogram and calibration diagram. **(A)** The nomogram developed in the present study; **(B)** Calibration curve of the derivation set. **(C)** Calibration curve of the validation set. SBP indicates systolic blood pressure; OTR indicates time from symptom onset to recanalization; ICH indicates intracranial hemorrhage.

**Figure 4:**
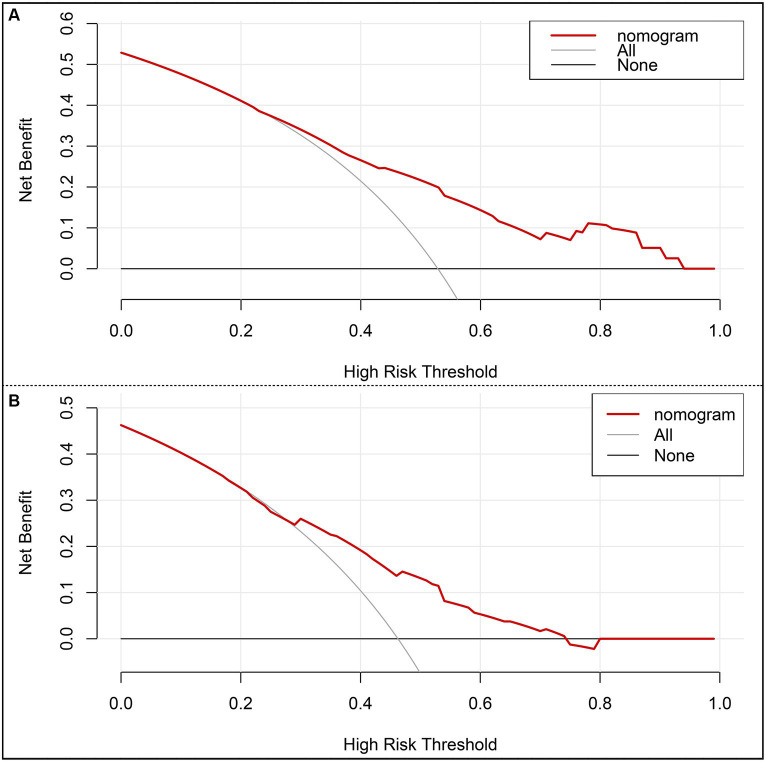
Decision curve analysis (DCA) of the nomogram. **(A)** DCA of the training set. **(B)** DCA of the internal validation set. x-axis, the threshold probability; y-axis, the net benefit.

The risk prediction nomogram of FR in AIS based on multivariate Logistic analysis is shown in Figure 3A, and each independent risk factor was assigned with a score ranging from 0 to 100 points. The four independent predictors obtained by multivariate analysis were scored according to the weight: when age ≥ 65, 43 points; When SBP ≥ 180 mmHg, 100 points; OTR ≥ 453 min, 59 points; When 24 h ICH occurs, 81 points, the final total score is 260 points. Thus, it can be concluded that there is a 50% probability of FR occurring when the total score is 73 (Figure 3A). For the model, after the internal verification of 1,000 bootstrap samples, the calibration diagrams of the derivation set (Figure 3B) and the validation set revealed that the nomogram model had good prediction accuracy (Hosmer-Lemeshow test: the derivation set X^2^ = 3.348, *p* = 0.764; the validation set X^2^ = 4.892, *p* = 0.429). The C-index of FR in the derivation set and the validation set were 0.739 (95%CI 0.662~0.816, st = 0.039; Figure 3B) and 0.703 (95%CI 0.621~0.785, st = 0.042; Figure 3C), respectively, suggesting a remarkable sensitivity and specificity of the nomogram in the clinical context. In addition, to evaluate the net benefit of the nomogram model in predicting a FR in AIS after MT, the DCA was performed. The results shown that when threshold probabilities are ranged between 25% and 93% in the derivation set (Figure 4A) and between 28% and 74% in the validation set (Figure 4B), using a nomogram to predict FR may have a greater net benefit than a “treat all” or “treat none” strategy, suggesting that the nomogram model has good clinical utility.

### Translation of the nomogram model into practice

**Figure 5:**
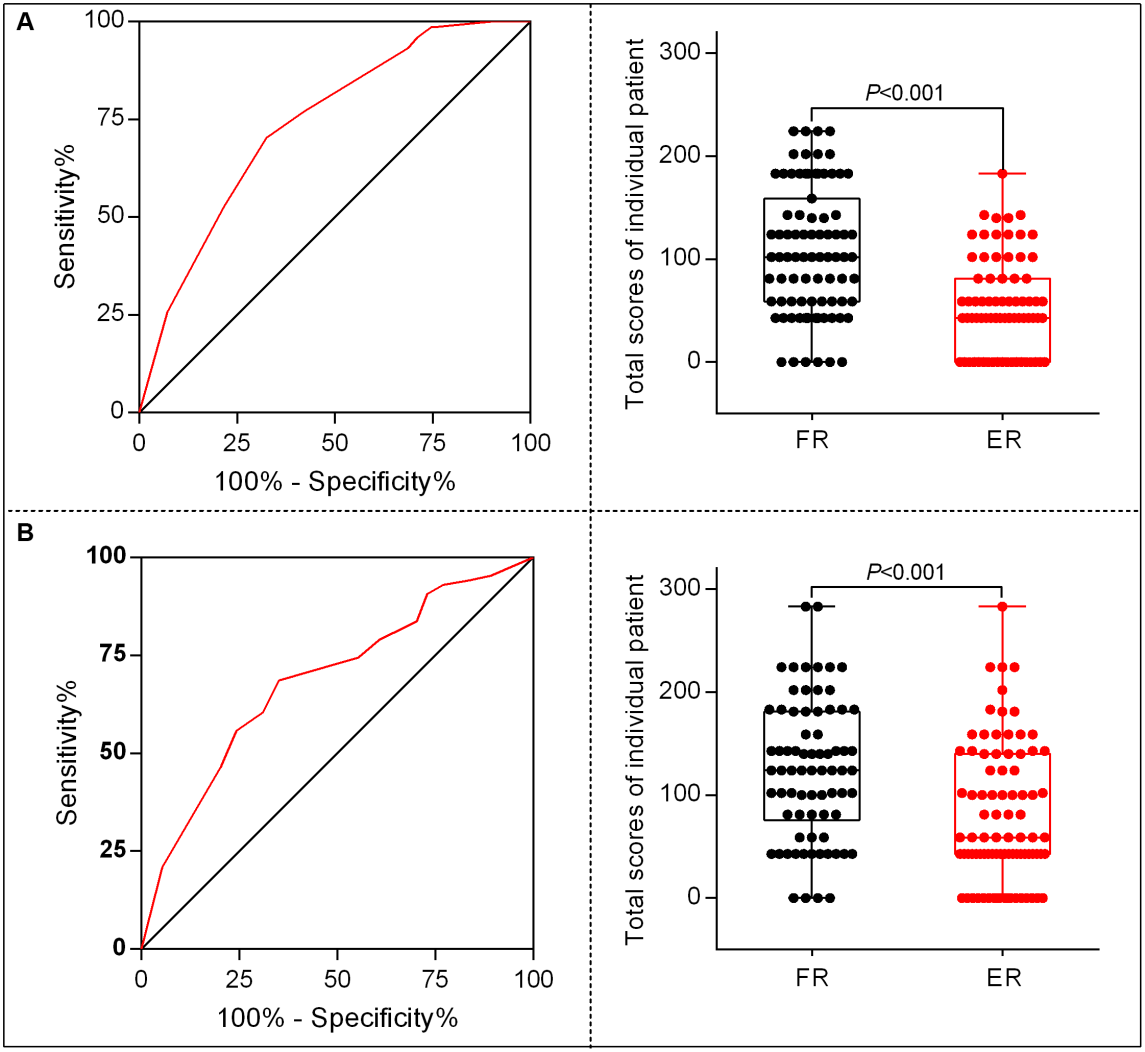
The characteristic curves of the nomogram model. **(A)** Receiver operating characteristics (ROC) for identifying the optimal threshold (left) and the scatter plot of the total scores (right) in AIS patients undergoing MT in the derivation set. **(B)** ROC for identifying the optimal threshold (left) and scatter plot of the total scores (right) in AIS patients undergoing MT in the validation set.

According to the points of each variable contributed to the nomogram, we translated the predictive model into practice. The total scores of each patient was calculated as follows: total scores = (0 for age < 65 or 1for age ≥ 65) × 43 + (0 for SBP < 180 mmHg or 1 for SBP ≥ 180 mmHg) × 100+ (0 for OTR < 453 min or 1for OTR ≥ 453 min) × 59 + (0 for 24 h no-ICH or 1for 24 h ICH occurs) × 81. The ROC curve and the scatter plot of the total scores of each patient in the derivation and the validation sets are shown in Figures 5A,B.

## Discussion

In this study, we explored the application of a nomogram to predict the probability of FR after MT treatment in AIS patients. Our nomogram incorporated variables, including age, preoperative SBP, OTR, and 24 h ICH, and showed good discriminatory ability, calibration, and value in clinical application after independent evaluation in the derivation and validation set.

Nomograms are highly reliable and practical estimation tools that help predict prognosis and enhance clinical decisions on personalized treatment by combining different prognostic and determinant data and evaluating them in combination with several powerful event indicators (9–11). They are also widely used to predict the prognosis of stroke, including FR (6, 12). Based on radiomics and with the help of machine learning, Yuqi Luo et al. established nomogram to predict FR, focusing on the use of machine learning methods to integrate clinical information and imaging information, which is complex and limits its extensional generalizability (5). ShiTeng Lin et al. reported a visualized nomogram to predict FR after endovascular thrombectomy in basilar artery occlusion stroke and Jincheng Guan et al. constructed nomogram-based prediction of the FR in AIS before and after endovascular therapy (6, 12). Jincheng Guan and ShiTeng Lin’s studies were all retrospective studies and with small sample sizes of 101 and 84 participants, respectively. In addition, the nomogram of ShiTeng Lin et al. was based on basilar artery occlusion stroke, and the model has not been validated. Jincheng Guan et al.’s model was a single-center retrospective study and was only validated internally, not externally, which was subject to selectivity bias and insufficient reliability. H Ni and H Wu et al. also reported predictors of FR in patients with intracranial atherosclerosis-related stroke, respectively, but neither of them built predictive models (13, 14). In this study, our model was aimed at intracranial internal carotid artery, MCA trunk acute obstruction in patients, and the data used for the modeling were from rigorous RCT studies, and the validation was an independent external validation. The derivation set strictly followed the RCT, and the FR prediction model obtained through statistical analysis had good calibration and differentiation. Data from the real world can reflect a broader and representative patient population, and also provide a series of supplementary evidence for RCT research (15, 16). To our knowledge, there have been no studies using real-world data as an external validation group for FR predictive models. In this study, our FR prediction model was further validated in a validation set from a real-world population, suggesting the reliability of the model.

Many studies have found that age is one of the potential risk factors for FR after MT in AIS patients (17). Consistent with their conclusions, this study also confirmed advanced age as an independent risk factor for FR, and on this basis further subdivided the age into AIS patients ≥ 65 years old.

Whether patients with AIS receive hypotensive treatment have always been a controversial issue. Studies have shown that higher SBP in AIS patients upon admission may be associated with good prognosis (18). Another study pointed out that there is a U-shaped relationship between the average SBP during hospitalization and the 90d prognosis of AIS patients (19), that is, patients have the best prognosis when the blood pressure is maintained between 135 and 150 mmHg, while patients have a worse prognosis when the blood pressure exceeds the above limit. At the same time, lower SBP may be associated with better prognosis in patients undergoing intravenous thrombolysis (17). In our study, it was found that preoperative SBP ≥ 180 mmHg was an independent risk factor for FR when AIS patients underwent MT. This finding can provide references for clinicians on whether and to what extent they should choose to reduce blood pressure during MT.

Previous studies (8) have proved that OTR in AIS patients undergoing MT therapy will seriously affect the probability of FR occurrence, which is consistent with our study. At the same time, this study further took 453 min as the boundary, and listed OTR ≥ 453 min as a risk factor for FR, providing clinicians with more accurate operation timing.

sICH is considered to be one of the risk factors for FR after intravascular therapy in AIS patients (8). However, our results suggest that 24 h sICH can not constitute an independent risk factor for predicting FR, whereas 24 h ICH is an independent risk factor for FR. This suggests that clinicians should pay more attention to patients with 24 h ICH, not just those with 24 h sICH.

There is a big difference between the results of this study and previous studies, such as NIHSS score, ASPECTS score and other indicators did not show significant statistical significance in our study. Although these indicators were included in the model establishment criteria after univariable analysis, unfortunately, these indicators were not meaningful in the final model. Therefore, this requires further validation in large-scale studies.

### Limitation

There are still some limitations in this study. First, although the data in our study came from multiple centers, it only included Asian populations and may lack generality. Secondly, the sample size of this study is limited, and high-quality large-scale RCT studies are still needed for further verification in the future. Third, missing samples in data collection may cause the accuracy of the model to be affected. Fourth, this study only included patients with internal carotid artery and middle cerebral artery occlusion, which may not be applicable to patients with posterior circulation occlusion. Fifth, Because the majority of enrolled patients were within the thrombectomy time window, the study design at that time did not use advanced imaging assessment to assess the ischemic penumbra, which was one of the limitations of this study.

## Conclusion

In conclusion, the current study developed a nomogram based on age, SBP, OTR, and 24 h ICH that plays a convincing role in evaluation of the risk of FR in AIS patients undergoing MT, which may benefit the guidance of the decision-making in individual patients to avoid FR. Nonetheless, further study with large-scale prospective and multiple centers are essential to verify our conclusions.

## Data availability statement

The original contributions presented in the study are included in the article/Supplementary material, further inquiries can be directed to the corresponding authors.

## Ethics statement

This study was reviewed and approved by the Local Ethics Committee of PLA Rocket Force Characteristic Medical Center (X2017008). The studies were conducted in accordance with the local legislation and institutional requirements. Written informed consent for participation was not required from the participants or the participants’ legal guardians/next of kin in accordance with the national legislation and institutional requirements.

## Author contributions

C-cL: Conceptualization, Data curation, Formal analysis, Writing – original draft, Writing – review & editing. Y-dY: Data curation, Formal analysis, Investigation, Writing – original draft. XL: Investigation, Methodology, Resources, Writing – original draft. A-fL: Investigation, Methodology, Software, Visualization, Writing – original draft. CL: Investigation, Software, Writing – original draft. Y-eL: Investigation, Methodology, Writing – original draft. C-cJ: Software, Writing – original draft. Y-yZ: Methodology, Writing – original draft. MJ: Funding acquisition, Investigation, Methodology, Project administration, Resources, Writing – review & editing. JL: Conceptualization, Data curation, Funding acquisition, Project administration, Software, Supervision, Writing – review & editing. W-jJ: Conceptualization, Funding acquisition, Methodology, Project administration, Resources, Supervision, Validation, Visualization, Writing – review & editing.
