# Supplementary material for: A novel nomogram to predict futile recanalization in patients with acute ischemic stroke undergoing mechanical thrombectomy
Source: Front Neurol. 2024 Mar 22;15:1367950. doi: 10.3389/fneur.2024.1367950 (PMC10995219; doi:10.3389/fneur.2024.1367950)
Supplement: Supplementary file 1 [file Table_1.docx]

Supplementary Table 1 Baseline characteristics of patients with successful recanalization after endovascular treatment

| Variables | | Derivation set  % (n); IQR (n=162) | Validation set  % (n); IQR (n=170) |
| --- | --- | --- | --- |
| Demographics | |  |  |
| Age (years) (median, IQR) | | 66 IQR (56～74) | 67 IQR (59～75) |
| ≥65 | | 59.3% (96/162) | 56.5% (96/170) |
| ＜65 | | 40.7% (66/162) | 43.5% (74/170) |
| Sex | |  |  |
| male | | 58.6% (95/162) | 52.9% (90/170) |
| female | | 41.4% (67/162) | 47.1% (80/170) |
| Medical history | |  |  |
| Hypertension | | 58.6% (95/162) | 58.8% (100/170) |
| Diabetes mellitus | | 23.5% (38/162) | 19.4% (33/170) |
| Coronary heart disease | | 17.3% (28/162) | 17.6% (30/170) |
| Atrial fibrillation | | 49.4% (80/162) | 46.5% (79/170) |
| Previous Stroke | | 11.7% (19/162) | 16.5% (28/170) |
| Baseline data | |  |  |
| Pre mRS Score | |  |  |
| =0 | | 94.4% (153/162) | 81.8% (139/170) |
| ≠0 | | 5.6% (9/162) | 18.2% (31/170) |
| Glucose(mmol/L) (median, IQR) | | 6.91 IQR (5.92～9.0) | 7.00 IQR (6.12～8.49) |
| ≥6.91 | | 50.0% (81/162) | 52.3% (79/151) |
| ＜6.91 | | 50.0% (81/162) | 47.7% (72/151) |
| Systolic Blood Pressure(mmHg) | | 138 IQR (124～158) | 142 IQR (130～160) |
| ≥180 | | 8.6% (14/162) | 11.7% (19/162) |
| ＜180 | | 91.4% (148/162) | 88.3% (143/162) |
| Diastolic Blood Pressure(mmHg) | | 82 IQR (75～90) | 89 IQR (77～96) |
| ≥101 | | 10.5% (17/162) | 19.1% (31/162) |
| ＜101 | | 89.5% (145/162) | 80.9% (131/162) |
| Initial NIHSS (median, IQR) | | 14.0 IQR (11～18) | 16 IQR (12～22) |
| ≥19 | | 23.5% (38/162) | 35.3% (60/170) |
| ＜19 | | 76.5% (124/162) | 64.7% (110/170) |
| ASPECT | 10 | 34.6% (56/162) | 23.5% (40/170) |
| 9 | | 16.0% (26/162) | 27.1% (46/170) |
| 8 | | 27.8% (45/162) | 33.5% (57/170) |
| 7 | | 21.6% (35/162) | 15.9% (27/170) |
| TOAST | Atheroma | 18.5% (30/162) | 51.2% (87/170) |
| Cardioembolic | | 81.5% (132/162) | 40.0% (68/170) |
| Dissection | | NA | 2.4% (4/170) |
| Others | | NA | 6.5% (11/170) |
| Intravenous thrombolysis | | 32.1% (52/162) | 34.1% (58/170) |
| First pass effect | | 40.1% (65/162) | 44.7% (76/170) |
| General anesthesia | | 48.1% (78/162) | 50.6% (86/170) |
| Location of lesions | |  |  |
| M1 | | 71.6% (116 /162) | 55.9% (95/170) |
| ICA | | 28.4% (46/162) | 44.1% (75/170) |
| Residual severe stenosis | | 15.4% (25/162) | 16.5% (28/170) |
| Rescue therapy | | 21.0% (34/162) | 15.9% (27/170) |
| Anticoagulant therapy | | 56.2% (91/162) | 10.0% (17/170) |
| OTP (median, IQR) | | 304 IQR (224～380) | 291 IQR (193～370) |
| ≥391min | | 22.2% (36/162) | 18.2% (31/170) |
| ＜391min | | 77.8% (126/162) | 81.8% (139/170) |
| OTR (median, IQR) | | 410 IQR (319～490) | 364 IQR (256～448) |
| ≥453min | | 37.7% (61/162) | 24.3% (41/169) |
| ＜453min | | 62.3% (102/162) | 75.7% (128/169) |
| Complications | |  |  |
| 24h ICH | | 31.5% (51/157) | 31.2% (53/170) |
| 24h sICH | | 4.9% (8/157) | 12.4% (21/170) |
| Outcomes | |  |  |
|  | |  |  |
| Futile recanalization(90D mRS≥3) | | 54.0% (88/162) | 48.2% (82/170) |
| 90D mortality | | 21.0% (34/162) | 12.4% (21/170) |

MT, mechanical thrombectomy; NIHSS, National Institute of Health stroke scale; ASPECT, Alberta Stroke Program Early CT Score; M1, Middle cerebral artery 1; mRS, modified Rankin Scale; ICH, intracranial hemorrhage; sICH, symptomatic intracranial hemorrhage; END, Early neurological deterioration. OTA, time from onset to admission; OTP, time from onset to puncture; OTR, time from onset to recanalization.
